# Parent Experience in Neonatal Hospitalization in Poland: A Cross-Sectional Pilot Study Using NSS-8 and PEC Frameworks

**DOI:** 10.3390/jcm14217486

**Published:** 2025-10-22

**Authors:** Oskar Komisarek, Maja Matthews-Kozanecka, Katarzyna Wiecheć, Tomasz Szczapa, Joanna Kasperkowicz, Teresa Matthews-Brzozowska, Przemysław Daroszewski, Włodzimierz Samborski, Ewa Mojs, Roksana Malak

**Affiliations:** 1 Department of Otolaryngology, Phoniatrics and Audiology, Faculty of Medicine, Collegium Medicum, Nicolaus Copernicus University in Toruń, 87-100 Toruń, Poland; 2 Department of Social Sciences and the Humanities, Poznań University of Medical Sciences, 60-806 Poznan, Poland; pszczolka-maja@o2.pl; 3 Department of Clinical Psychology, Poznań University of Medical Sciences, 60-812 Poznan, Poland; k.wiechec@interia.eu (K.W.); ewa_mojs@poczta.onet.pl (E.M.); 4 Neonatal Biophysical Monitoring and Cardiopulmonary Therapies Research Unit, II Department of Neonatology, Poznan University of Medical Sciences, 60-535 Poznan, Poland; tszczapa@gmail.com; 5 Department and Clinic of Rheumatology, Rehabilitation and Internal Medicine, Poznań University of Medical Sciences, 61-545 Poznan, Poland; joanna.monika.borek@gmail.com (J.K.); wlodzimierz.samborski@ump.edu.pl (W.S.); rmalak@ump.edu.pl (R.M.); 6 Department of Maxillofacial Orthopaedics and Orthodontics, Collegium Medicum, Nicolaus Copernicus University in Toruń, Jagiellońska 13-15, 85-067 Bydgoszcz, Poland; 7 Department of Organization and Management in Health Care, Poznan University of Medical Sciences, 61-545 Poznan, Poland; dyrektor@orsk.pl

**Keywords:** neonatal intensive care, parent-reported experience, PREMs, family-centred care, clinical communication, NSS-8, PEC, discharge communication bundle, caregiver stress, quality improvement, post-discharge follow-up, Poland, pilot study

## Abstract

**Background:** Parent-reported experience in neonatal units is a key but under-measured dimension of family-centred care in Poland. We piloted a brief parent-experience questionnaire informed by the Neonatal Satisfaction Survey (NSS-8) and communication constructs from the Parents’ Experiences of Communication in Neonatal Care (PEC) to describe in-hospital experience and identify actionable targets for improvement. **Methods:** Observational, cross-sectional pilot at a Polish tertiary centre (September–November 2021). Parents of hospitalized neonates completed a 21-item survey covering educational materials, medical care/communication, parental stress/confidence, hospitalization details, and sociodemographics. Analyses were descriptive with item-wise denominators (*n* = 32–46). **Results:** Forty-six parents participated. Educational materials were rated very highly: parental guide 9.8/10 (*n* = 46); brochure readability 10/10 (*n* = 46), indicating ceiling effects. Perceptions of care and communication were favourable: overall care 4.47/5, physician concern 4.62/5, ward conditions 4.47/5, explanation of test indications 4.23/5, and adequacy/understandability of information 4.35/5 (each *n* = 35; medians = 5). Despite this, parental stress/anger/insomnia was moderate (3.00/5; *n* = 35), while confidence in basic home care remained high (4.10/5; *n* = 35). Following discharge, 17/46 (37.0%) sought specialist consultations. Length of stay (*n* = 34) had a median of 1 day (0–4). Reasons for admission most frequently included multisymptom presentations (20/46, 43.5%); respiratory (8.7%) and infectious (6.5%) causes were less common. **Conclusions:** Parents reported very positive care and communication alongside persistent emotional burden and substantial post-discharge information needs. Findings support pairing a broad experience framework with a focused communication module, standardizing discharge communication (including a 48–72 h “bridging” contact), and progressing to a multicentre psychometric validation. This exploratory pilot was not a formal validation study; mixed scales and item-wise missingness should guide instrument refinement.

## 1. Introduction

Hospitalization of newborns in tertiary neonatal units is consistently reported by parents as stressful, with uncertainty and sleep disturbance often extending beyond discharge. Parental wellbeing shapes early caregiving, adherence to follow-up, and the family’s capacity to navigate complex care, underscoring the need to complement biomedical stabilization with family-centred approaches [1,2,3,4]. In Europe, recent syntheses indicate that preterm birth (PTB) rates remain substantial and relatively stable, with wide between-country variation and persistent societal and health-economic burdens despite clinical advances [5,6]. This epidemiologic backdrop reinforces the importance of improving not only outcomes for neonates but also the experience of care for families.

At the same time, pathways for predicting and preventing PTB remain heterogeneous across health systems, from variation in cervical-length screening to selective adoption of biomarker-guided strategies; no single algorithm captures most PTB cases, and implementation is inconsistent [5]. Such system-level heterogeneity limits the interpretability of purely clinical quality indicators and elevates the value of parent-reported experience and bedside communication quality as actionable levers for improvement. We use parent-reported experience measures (PREMs), i.e., brief questionnaires that capture parents’ experience with care processes, communication, and the care environment. Communication cadence, modality and clarity are particularly amenable to iterative quality-improvement cycles and may influence how families interpret risk, participate in decisions and prepare for discharge [7,8,9].

Multiple instruments are available to measure parent experience in neonatal care. Broad, multi-domain tools such as EMPATHIC-N capture overall satisfaction but are commonly administered post-discharge, offering limited granularity on real-time communication at the cot side [7,8]. In contrast, communication-focused tools—e.g., Parents’ Experiences of Communication in Neonatal Care (PEC)—foreground the immediacy, comprehensibility and frequency of information exchange [9]. Complementing these, the Neonatal Satisfaction Survey (NSS-8) provides an eight-domain framework spanning care/treatment, information, ward environment and parental anxiety, with demonstrated internal consistency and construct coherence [10,11]. Collectively, these instruments highlight that how and how often information is delivered can be at least as salient for parents as what is conveyed.

However, operational, in-hospital data from Poland describing parent experience and communication-specific performance measures remain scarce. This gap limits the ability of Polish units to benchmark, to close feedback loops during hospitalization and to translate “global satisfaction” scores into concrete, unit-level actions; without locally generated data, it is difficult to determine whether international findings generalize to the Polish context or where implementation barriers may differ [12]. Although validated instruments such as NSS-8 and PEC exist, no Polish version or culturally adapted equivalent has been validated. Communication practices and organizational structures differ across health systems; therefore, this pilot aimed to design and preliminarily test a linguistically and contextually appropriate questionnaire informed by both frameworks to support future multicentre validation.

Study aim. In response, we conducted a single-centre pilot study in Poland to develop and field-test a brief, pragmatic parent-experience questionnaire informed by NSS-8 domains and communication constructs from PEC. Our primary objective was to characterize parent-reported experience during hospitalization in a Polish tertiary neonatal unit and to identify priority targets for improvement (information adequacy/clarity, ward environment/visits, parental anxiety). Secondary objectives were to assess feasibility and acceptability of near-real-time feedback and to generate implementation insights for future multicentre work (e.g., discharge-communication bundles). We explicitly frame this as an exploratory pilot rather than a psychometric validation. Based on prior literature, we hypothesized a characteristic asymmetry: generally favourable ratings of clinical care coexisting with meaningful emotional burden among parents [9,10,11].

## 2. Materials and Methods

This observational, cross-sectional pilot study was carried out at the Gynecology and Obstetrics Clinical Hospital of the Poznan University of Medical Sciences (Poland). The participating unit functions as a tertiary perinatal and neonatal ward admitting both preterm and term neonates for a variety of medical or observational reasons. The cohort therefore reflects the general neonatal population of a tertiary centre rather than an intensive care cohort of extremely low birth weight neonates. Data were collected between September 2021 and November 2021. The study protocol was approved by the Bioethics Committee (Approval No. 481/21, issued 21 June 2021). All procedures complied with the Declaration of Helsinki. Written informed consent was obtained from all participants before completing the questionnaire. This study was not prospectively registered in any publicly accessible registry such as ClinicalTrials.gov or PROSPERO, as it was an observational, cross-sectional pilot study.

Forty-six parents of hospitalized neonates participated. Parents were approached during their child’s hospital stay or shortly after discharge and invited to complete the questionnaire. This approach was intentional to capture both real-time in-hospital experiences and immediate post-discharge reflections. The timing of completion was recorded but not used for stratified analysis due to the small sample size. Inclusion criteria were: (1) parent or legal guardian of a neonate or infant hospitalized at the neonatal or paediatric ward; (2) ability to read and complete the questionnaire in Polish; and (3) provision of informed consent. Exclusion criteria were lack of consent or a questionnaire without usable responses. Not all participants answered every item; consequently, denominators vary across analyses (item-wise *n* = 32–46). Missing demographic information such as parity reflected incomplete questionnaire responses rather than absence of clinical records. Clinical data were not extracted from hospital charts to preserve anonymity, as the survey was fully parent-reported. This missingness may affect the representativeness of some item-level results and is acknowledged as a limitation. Recruitment flow. All parents of hospitalized neonates and neonates during the study period were eligible for participation. While 46 questionnaires were completed and included in the analysis, detailed information on the total number of parents approached, refusals, or incomplete responses was not systematically recorded. Because detailed data on the number of parents approached, refusals, and incomplete responses were not systematically collected, a formal recruitment flowchart could not be generated. This limitation restricts the assessment of potential non-response bias and will be addressed in future multicentre validation studies through systematic participant tracking and flowchart reporting.

The survey included 21 items (18 structured questions and 3 demographic questions) organized into five domains:

1. Educational materials—assessment of the parental guide and brochure (0–10 scale);
2. Medical care and communication—evaluation of treatment, physician concern, ward conditions and adequacy of explanations (1–5 Likert scales);
3. Parental stress and confidence—stress related to hospitalization and confidence in providing basic childcare at home (1–5 Likert scales);
4. Hospitalization details—gestational age, length of stay, reasons for hospitalization (multiple choice) and history of previous admissions;
5. Sociodemographics—number of children, respondent’s age, respondent identity (mother, father, other) and travel time to hospital.

The questionnaire was developed specifically for this project and was not psychometrically validated; therefore, this analysis should be interpreted as a pilot exploring feasibility and potential clinical utility.

The questionnaire was developed specifically for this study based on domains from the NSS-8 and PEC tools. The item pool was conceptually structured around two validated frameworks: the Neonatal Satisfaction Survey (NSS-8) and the Parents’ Experiences of Communication in Neonatal Care (PEC). Items reflecting care and treatment quality, adequacy of information, ward environment, and parental anxiety were derived from NSS-8 domains. Items addressing the clarity, adequacy, and frequency of physician–parent communication were adapted from PEC constructs. In addition, several context-specific questions were developed de novo to reflect the Polish clinical and organizational setting, including readability of educational materials, parental confidence in basic childcare at home, and post-discharge contact or follow-up needs. This mapping ensured conceptual continuity with existing instruments while allowing contextual adaptation to local neonatal care practices. A detailed mapping of each questionnaire item to its conceptual source (NSS-8, PEC, or newly developed) is provided in Supplementary Table S1. Initial items were drafted by the study team and reviewed by a panel of clinicians, communication experts, and neonatal researchers, including co-authors of the present paper. The expert panel assessed content validity, clarity of wording, and clinical relevance. This pilot study served as the first field test of the instrument in the target population. The average time to complete the questionnaire was not formally measured.

Analyses were primarily descriptive. Continuous variables are presented as means, medians, minima and maxima. Categorical variables are summarized as counts and percentages. For multiple-response questions (e.g., reasons for hospitalization), each option was analyzed separately and percentages were calculated relative to the total sample (*N* = 46). Given the pilot nature and small sample size, no advanced statistical modelling was performed. Data analysis was conducted using Microsoft Excel (Microsoft Corp., Redmond, WA, USA). Data handling and missingness. Data were analysed item-wise with explicit denominators (*n* = 32–46) to account for partial non-response. No imputation was performed. The proportion of missing responses is reported for each item in Table 1 and Table 2. One implausible gestational age value (“1 week”) was identified and excluded from analysis in accordance with a predefined plausibility rule.

## 3. Results

All 46 parents completed the questionnaire. Median respondent age was 31.5 years (*n* = 32, range 18–44). The respondent was the mother in 30/46 (65.2%), father in 5/46 (10.9%) and other/missing in 11/46 (23.9%). Gestational age at birth was available for 34/46 respondents (median 36.5 weeks; one record miscoded as “1 week”; excluded from range reporting). Parity data were available for 34/46 (median 1 child, range 1–3). Item-wise denominators for all outcomes are reported below and in Table 1.

Evaluation of educational materials

The parental guide was rated 9.8/10 (*n* = 46, median 10, range 7–10), and brochure readability 10/10 (*n* = 46, median 10, range 9–10), indicating marked ceiling effects.

Medical care and communication

Overall quality of care/treatment was 4.47/5 (*n* = 35, median 5). Physician concern was 4.62/5 (*n* = 35, median 5). Ward conditions during visits were 4.47/5 (*n* = 35, median 5). Reasons for examinations were reported as well explained (mean 4.23/5, *n* = 35, median 5), and information as adequate/understandable (mean 4.35/5, *n* = 35, median 5). Distributions and item-wise denominators are provided in Table 1; a high share of maximum-score responses suggests potential ceiling effects for these items.

Parental stress and confidence

Stress/anger/insomnia related to hospitalization averaged 3.00/5 (*n* = 35, median 3; higher = more stress). Confidence in basic childcare at home was 4.10/5 (*n* = 35, median 4). These results indicate moderate stress despite high confidence. The combination of moderate stress scores and post-discharge consultations indicates informational gaps and unmet needs regarding discharge communication and early follow-up.

Hospitalization characteristics

The most frequently selected reason for admission was multiple concurrent or nonspecific clinical concerns (20/46, 43.5%), most often including feeding difficulties, irritability, or poor postnatal weight gain not attributable to a single diagnosis. Other reported reasons included respiratory problems (4/46, 8.7%), infections (3/46, 6.5%), glucose disturbances (2/46, 4.3%), and isolated poor weight gain (1/46, 2.2%); no seizures, phototherapy, or cardiac problems were reported. Reasons for hospitalization are detailed in Table 2. Length of stay was available for 34/46 (median 1 day, range 0–4). Prior hospitalization of the child was reported by 12/34 (35.3%), with 22/34 (64.7%) reporting none (denominator restricted to respondents with available data). Regarding visitation, 13/46 (28.3%) reported being the sole family member allowed to visit and 16/46 (34.8%) reported shared visitation (17/46 [37.0%] missing/other). Only 7/46 (15.2%) reported receiving help from family/friends; 11/46 (23.9%) were missing/unclear. Travel time was recorded as a categorical code (0–4) rather than absolute minutes. Full descriptive characteristics, including item-level distributions for all sociodemographic and experience variables, are presented in Table 1.

Follow up consultations

After discharge, 17/46 (37.0%) sought additional specialist consultations, indicating that a substantial share of families pursued further evaluation post-hospitalization.

Sensitivity/feasibility notes

Item-wise response counts ranged 32–46, reflecting partial non-response. Analyses were performed without imputation, with denominators reported per item. High rates of maximum scores for several items suggest limited discriminatory range and will inform subsequent instrument refinement.

## 4. Discussion

In this single-centre Polish pilot, we field-tested a brief parent-experience questionnaire informed by the domains of the Neonatal Satisfaction Survey (NSS-8) and communication constructs described in PEC studies, rather than conducting a formal application or validation of the NSS-8 instrument itself. This pilot did not aim to compare or replace existing instruments but to explore feasibility and content relevance of an adapted tool derived from both frameworks in the Polish context. Parents reported very high satisfaction with educational materials and clinical care, while moderate stress during hospitalization persisted. Additionally, 37% sought specialist consultations after discharge, suggesting residual informational or emotional needs despite favourable in-unit ratings. Given item-level denominators (*n* = 32–46) and multi-response items, all inferences are exploratory.

Our pattern—high ratings of care and information coexisting with notable parental stress—is broadly consistent with literature using multi-domain satisfaction frameworks such as NSS-8, which emphasize care/treatment, information, ward environment and parental anxiety [10,11]. Prior analyses have linked higher satisfaction to communication quality, social support and involvement in decisions [11]. In our cohort, adequacy and clarity of explanations scored highly, yet stress did not fully resolve, aligning with reports that favourable care ratings can co-occur with psychological burden when contextual supports are limited [10,11]. Ceiling effects observed across several items highlight a need for greater response variability, a phenomenon also noted in validation studies of similar instruments [7,8,9].

Communication-focused evidence helps interpret these findings. Studies developing and adapting PEC highlight the salience of cadence, modality and comprehensibility of updates and demonstrate the feasibility of near-real-time measurement using structured translation procedures, expert review and cognitive interviewing [9]. While broad tools provide a global signal, coupling them with a targeted communication module may sharpen interpretability and point to concrete, testable changes (e.g., structured update schedules, standardized discharge messages) [7,8,9].

By combining items conceptually aligned with established NSS-8 and PEC frameworks with several newly developed, context-specific questions, this pilot aimed to balance international comparability with national relevance. Such hybrid design enables continuity with prior validation studies while capturing culturally and organizationally distinct features of Polish neonatal care. This approach supports the development of a fully validated Polish parent-experience instrument that preserves cross-national conceptual integrity but remains sensitive to local communication norms and healthcare structures.

The post-discharge interface emerged as a pragmatic target. Based on these findings, key improvement priorities include the implementation of standardized discharge communication bundles, clear post-discharge contact channels, and structured parental counselling during the transition home. Families frequently report barriers in early primary-care encounters—limited time, inconsistent advice—whereas clear, timely, empathic counselling supported by written resources functions as a facilitator. The observed rate of post-discharge consultations in our pilot underscores this gap and supports implementing a discharge communication bundle (concise diagnosis/plan, red-flag symptoms, feeding/sleep guidance and a named contact route) plus an early 48–72 h bridging contact; evidence from parent-education and empowerment programmes suggests such interventions can reduce parental distress and improve outcomes [13,14,15,16,17,18].

### 4.1. Strengths and Limitations

Strengths include being, to our knowledge, the first Polish pilot to operationalize a parent-experience instrument informed by established domains; transparent reporting of item-wise denominators and multi-response handling; and an explicit focus on communication. However, several limitations temper interpretation. The sample was small and single-centre, fathers were under-represented and we did not perform psychometric analyses (e.g., internal consistency, test–retest, factor structure). The sample was small and single-centre, fathers were under-represented and we did not perform psychometric analyses (e.g., internal consistency, test–retest, factor structure). Because the cohort mainly comprised late-preterm and term neonates (median gestational age 36 weeks), the findings primarily represent parent experiences in general neonatal hospitalization rather than high-dependency intensive care of extremely preterm neonates. Mixed response scales (0–10 and 1–5) complicate cross-item comparisons and contributed to ceiling effects (e.g., materials rated at the maximum). Item-level missingness, including occasional gaps in basic demographic data such as parity, resulted from voluntary non-response on specific questions and not from lack of access to clinical information. Additionally, because recruitment flow data were not systematically recorded, the rate of non-participation could not be determined, which may limit interpretability regarding potential response bias. The limited sample size (*n* = 46) and single-centre recruitment restrict the generalizability of these findings. Fathers constituted only 10.9% of respondents, highlighting the need for gender-balanced recruitment in future multicentre studies with predefined subgroup analyses (e.g., mothers vs. fathers, preterm vs. term. Mixed response scales (0–10 and 1–5) complicate cross-item comparisons and contributed to ceiling effects (e.g., materials rated at the maximum). Surveys completed during or shortly after hospitalization may be prone to courtesy and social-desirability bias. Finally, variation in item-wise denominators and the need for clearer clinical categorization of ‘reason for admission’ underscore the importance of pre-specified codebooks and routine data checks. Finally, variation in item-wise denominators and the need for clearer clinical categorization of “reason for admission” underscore the importance of pre-specified codebooks and routine data checks. In particular, the term ‘multisymptom presentation’ used in this pilot reflected non-specific or overlapping neonatal symptoms reported by parents and clinicians; future multicentre studies will adopt standardized diagnostic categories to enhance comparability. Looking beyond the admission episode, future work should link parent experience to developmental follow-up using validated tools (e.g., Alberta Infant Motor Scale [19], Harris Infant Neuromotor Test [20]) and Polish unit-level assessments [12] to understand whether communication improvements translate into downstream functional benefits.

### 4.2. Implications and Next Steps

We propose three actionable directions for a learning-health-system loop:

1. Add a brief communication module (PEC-derived/adapted) to disentangle content, cadence and channel, with pre-registered item-performance criteria and iterative wording refinements [9].
2. Standardize discharge messaging via a concise bundle and a 48–72 h bridging contact; prospectively track process measures (bundle completeness, proportion reached within 72 h) and outcomes (reassurance, unplanned contacts) [18].
3. Scale to a multicentre study powered for psychometrics (internal consistency, test–retest, confirmatory factor analysis) and pre-specified subgroup analyses (mothers vs. fathers; preterm vs. term; length of stay; travel time; parity), co-measuring parental stress/anxiety and empowerment to test whether communication improvements mediate reductions in stress and gains in competence [13,14,15,16,17].

The findings provide preliminary, context-specific insights from a Polish tertiary setting and are intended to inform the design and implementation of a future multicentre validation study rather than to generate generalizable conclusions.

## 5. Conclusions

This pilot suggests that, within a Polish tertiary setting, parents perceive care and information very positively, yet emotional burden and post-discharge uncertainty remain. Pairing a broad experience framework with a targeted communication metric and standardizing discharge communication provides a practical path from description to actionable, family-centred quality improvement likely to benefit parental wellbeing and infant outcomes. Future research should therefore include larger, multicentre cohorts to ensure representativeness and gender diversity.

## Figures and Tables

**Table 1:** Descriptive characteristics and parent-reported experience.

Question	*N*	Mean	Median	Min	Max	Distribution/Notes
How do you assess the content of the parental brochure? (0–10)	46	9.8	10	7	10	
Was the brochure clear and easy to read? (0–10)	46	10.0	10	9	10	
Did you seek further specialist consultation after discharge?	46					Yes (1): 17/46 (37.0%); No (0): 29/46 (63.0%)
How do you assess the quality of care and treatment your child received? (1–5)	35	4.47	5	1	5	
Did the physician show concern for your child’s condition? (1–5)	35	4.62	5	3	5	
Satisfaction with ward conditions during the visit (1–5)	35	4.47	5	1	5	
Were reasons for examinations explained to you in an understandable way? (1–5)	35	4.23	5	1	5	
Were the explanations adequate and understandable? (1–5)	35	4.35	5	1	5	
To what extent did you experience stress/anger/worry during hospitalization? (1–5)	35	3.00	3	1	5	Higher = more stress
Do you feel confident in basic childcare at home? (1–5)	35	4.10	4	1	5	
Gestational week at birth	34	34.2	36.5	1 *	42	* One implausible value (1 week) identified and excluded in analysis. After exclusion: *N* = 33, mean = 35.2, median = 36.5, max = 42.
How many children have you given birth to?	34	1.15	1	1	3	
Why was your child hospitalized? (multiple answers possible)	46					See Table 2
Length of hospital stay (days)	34	1.56	1	0	4	
Have your children been hospitalized before?	34					Yes: 12/34 (35.3%); No: 22/34 (64.7%)
How do you assess your child’s current health now?	46					4: 10/46 (21.7%); 3: 7/46 (15.2%); 2: 12/46 (26.1%); 1: 4/46 (8.7%); Missing: 13/46 (28.3%)
Were you the sole family member allowed to visit?	34					Yes (1): 13 (28.3%); No (2): 16 (34.8%); Other: 5 (10.9%); Missing: 11 (23.9%)
Did you receive help from family/friends?	34					Yes (1): 7 (15.2%); No (2): 2 (4.3%); Other categories reported; Missing: 11 (23.9%)
Travel time from home to hospital (minutes)	34	1.82	1.41	0	4	Scale in categorical codes, not absolute minutes
Who completed the questionnaire?	46					Mother: 30/46 (65.2%); Father: 5/46 (10.9%); Other/Missing: 11/46 (23.9%)
Respondent age (years)	32	31.7	31.5	18	44	

* marks an implausible data entry (1 week) retained in the raw export but excluded from analysis; recalculated descriptive statistics after exclusion are provided in the notes. For categorical items, counts are shown as *n*/*N* with percentages in parentheses where applicable. ‘*N*’ indicates item-wise respondents unless stated otherwise.

**Table 2:** Reasons for hospitalization (multiple answers possible).

Reason for Hospitalization	*N* (%)
Multiple symptoms (syndrome of more than one issue)	20 (43.5%)
Respiratory problems	4 (8.7%)
Infection	3 (6.5%)
Glucose metabolism problems	2 (4.3%)
Poor weight gain	1 (2.2%)
Seizures	0 (0%)
Phototherapy	0 (0%)
Cardiac problems	0 (0%)
Missing/unclear data	4 (8.7%)

## Data Availability

Data are available only under the permission of authors and parents of neonates described in the manuscript.

## Supplementary Materials

The following supporting information can be downloaded at: https://www.mdpi.com/article/10.3390/jcm14217486/s1, Table S1: Conceptual origin and examples of questionnaire items.
