# Supplementary material for: Parent Experience in Neonatal Hospitalization in Poland: A Cross-Sectional Pilot Study Using NSS-8 and PEC Frameworks
Source: J Clin Med. 2025 Oct 22;14(21):7486. doi: 10.3390/jcm14217486 (PMC12610416; doi:10.3390/jcm14217486)
Supplement: Supplementary file 1 [file jcm-14-07486-s001.zip › jcm-3889990-supplementary.pdf]

Supplementary Table S1. Conceptual origin and examples of questionnaire items.

Domain	Conceptual Source	Construct Measured	Example Item (English translation)	Type
Care and treatment quality	NSS-8	Parental perception of care and treatment received	How do you assess the quality of care and treatment your child received?	Adapted
Physician concern	NSS-8	Professional attitude and empathy	Did the physician show concern for your child's condition?	Adapted
Ward environment	NSS-8	Physical and emotional conditions during visits	How do you assess ward conditions during the visit?	Adapted
Explanation of examinations	PEC	Clarity and comprehensibility of medical communication	Were reasons for examinations explained to you in an understandable way?	Adapted
Adequacy of information	PEC	Completeness and adequacy of information provided	Were the explanations adequate and understandable?	Adapted
Parental anxiety / stress	NSS-8	Emotional burden related to hospitalization	To what extent did you experience stress, anger, or worry during hospitalization?	Adapted
Confidence in basic childcare	Newly developed	Parental self-efficacy and readiness for home care	Do you feel confident in basic childcare at home?	New
Readability of educational materials	Newly developed	Clarity and linguistic accessibility of materials	Was the brochure clear and easy to read?	New
Evaluation of educational content	Newly developed	Perceived usefulness of written information	How do you assess the content of the parental brochure?	New
Post-discharge support	Newly developed	Continuity of care and informational needs after discharge	Did you seek further specialist consultation after discharge?	New
Sociodemographic information	Newly developed	Contextual variables for analysis (e.g., parental role, parity, travel time)	Who completed the questionnaire? / How many children have you given birth to?	New
